# Short-Term Functional Outcomes and Quality of Life after B2.1 Type Pelvic Fractures for Surgically and Non-Surgically Treated Young Patients

**DOI:** 10.3390/medicina57060513

**Published:** 2021-05-21

**Authors:** Giedrius Petryla, Valentinas Uvarovas, Rokas Bobina, Jaunius Kurtinaitis, Tomas Sveikata, Sigitas Ryliškis, Roma Puronaitė, Giedrius Kvederas, Igoris Šatkauskas

**Affiliations:** Clinic of Rheumatology, Orthopaedics Traumatology and Reconstructive Surgery, Faculty of Medicine, Vilnius University, LT-03101 Vilnius, Lithuania; giedrius.petryla@gmail.com (G.P.); valiusuvarovas@gmail.com (V.U.); jauniusk1@yahoo.com (J.K.); tomas.sveikata@mf.vu.lt (T.S.); ryliskis.s@gmail.com (S.R.); romantina@gmail.com (R.P.); giedrius.kvederas@santa.lt (G.K.); igoris.satkauskas@gmail.com (I.Š.)

**Keywords:** pelvic fracture, pelvic injury, functional outcomes, quality of life

## Abstract

*Background and Objectives*: Lateral compression injuries of the pelvic ring are most common among young and elderly patients. Of all pelvic ring fracture injuries, the B2.1 type—involving lateral compression of the pelvic ring—is the most common. Despite this, we still have no high-level evidence to consult when choosing between the surgical and non-operative approaches. The purpose of this research was to compare the short-term functional and quality of life outcomes between operatively and non-operatively treated young patients after a B2.1 type pelvic fracture. *Materials and Methods*: Patients aged 18 to 65 years with pelvic B2.1 type fractures—according to AO/Tile classification—that were hospitalized in a single trauma center between 2016 November and 2019 September were included in the research. Patients were retrospectively divided into two groups regarding their treatment: non-operative and operative. Functional outcomes were evaluated using Majeed score, and SF-36 was used for the evaluation of quality of life. Patients completed these questionnaires twice: first during hospitalization, regarding their pre-traumatic condition (timepoint I); and again 10 weeks after the injury, regarding their current condition (timepoint II). *Results*: A total of 55 patients (70.6% of whom were female) with type B2.1 pelvic fractures were included in the analysis, with an average age of 37.24 ± 13.78 years. There were 21 (38.18%) patients with high injury severity, and 37 (67.3%) patients were treated operatively versus 18 (32.7%) non-operatively. Between the two timepoints, Majeed score reduced by 34.08 ± 18.95 for operatively and 31.44 ± 14.41 for non-operatively treated patients. For operatively and non-operatively treated patients, the physical component summary (PCS) of the SF-36 questionnaire reduced by 19.45 ± 9.95 and 19.36 ± 7.88, respectively, while the mental component summary (MCS) reduced by 6.38 ± 11.04 and 7.23 ± 10.86, respectively. *Conclusions*: We observed that operative treatment of B2.1 type pelvic fractures for young patients is not superior to non-operative in the short-term, because the functional outcomes and quality of life are similar in both groups.

## 1. Introduction

The most common type of pelvic ring fractures are type B2.1, according to the AO/Tile classification, which are also known as lateral compression type 1 (LC-1) injuries, according to the Young and Burgess classification [1,2,3,4,5]. Lateral compression injuries of the pelvic ring are most common among young and elderly patients [6,7,8], and the prevalence of type B2 fractures ranges from 45% to 63% of all pelvic ring fractures [1,6,7,9,10]. Although lateral compression type B2.1 are the most common type of pelvic ring fractures, we still have no high-level evidence to consult when treating them. Most authors recommend non-operative treatment [2,3,4,11,12,13], yet the frequency of the surgical approach remains high—the German Pelvic Trauma Registry, for example, reported that the rate of operative treatment reached 24% of all type B2.1 pelvic fracture cases [13]. Furthermore, Tosounidis et al. reported that the number of operatively treated type B fractures increased from 31% to 40% over a period of 18 years [14]. The results of most studies investigating the long- and short-term functional outcomes of pelvic LC-1 fractures are inconsistent. The latest study, conducted by Höch et al., compared the outcomes of the non-operative and operative treatments of type B2.1 pelvic fractures, and showed that, although operatively treated patients had a significantly higher complication rate, there was no difference in other outcomes between groups [13].

We believe that the most important thing for young and working persons who sustain pelvic injuries is to return to active daily life, and work, as soon as possible. This is why long-term outcomes are not the only important consideration. To date, there have been no studies that provide high-level evidence comparing the short-term outcomes of operative and non-operative treatments of type B2.1 pelvic fractures. The aim of this study was to compare the short-term outcomes of pelvic function and quality of life between the operative and non-operative treatment of type B2.1 pelvic fractures in young patients. Our hypothesis was that the short-term functional outcomes and quality of life of non-operatively treated patients would be demonstrably inferior.

## 2. Materials and Methods

This investigation involved a single-center cohort study that considered the short-term outcomes of pelvic function and quality of life in both surgically and non-surgically treated young patients who had suffered an B2.1 pelvic injury. This study was performed in accordance with the ethical standards of the Vilnius Regional Biomedical Research Ethics Committee (approval No. 158200-16-868-394, 4 November 2016) and complied with the 1964 Declaration of Helsinki and its later amendments, or comparable ethical standards. Written informed consent was obtained from each study participant.

**Figure 1:**
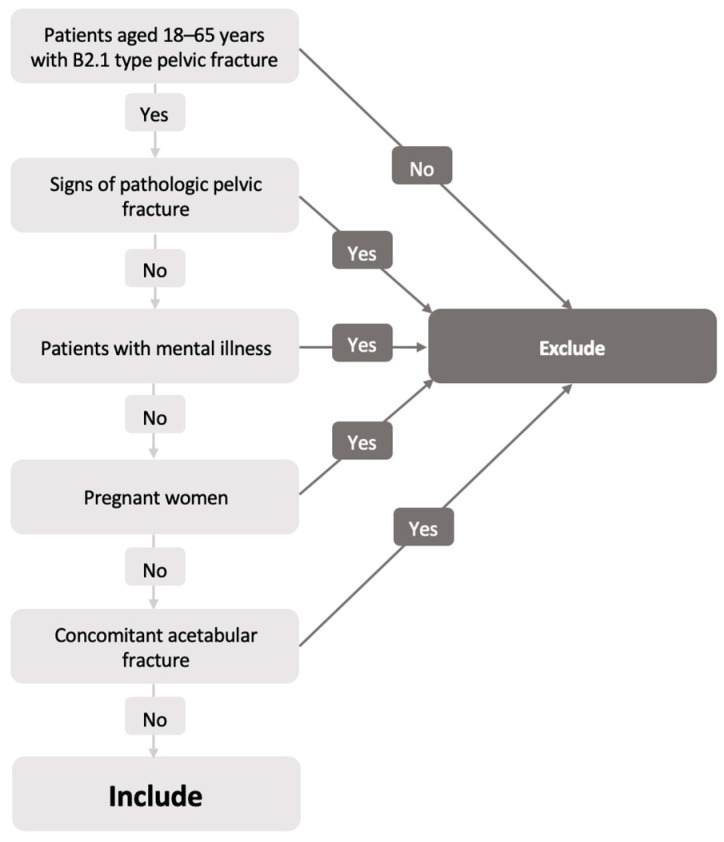
Flowchart demonstrating the inclusion criteria of the patients.

Patients aged 18 to 65 years with pelvic B2.1 type fractures that were hospitalized in a single trauma center between 2016 November and 2019 September were included in the research. Patients older than 65 years or with pathologic pelvic fractures, pregnant women, patients with mental illnesses, and those with a concomitant acetabular fracture were excluded (Figure 1).

Pelvic radiography and computed tomography (CT) were performed for each patient. Fractures were classified according to the AO/OTA pelvic fracture classification by two independent senior radiologists.

The personal data of each patient was collected, including: gender; age; date of trauma; Injury Severity Score (ISS); type of treatment (non-operative or operative); concomitant injuries; and surgeries. High injury severity was diagnosed with the threshold of ISS ≥ 18 [15,16].

For the analysis of the data collected, patients were retrospectively divided into two groups regarding their treatment: non-operative; and operative. The main criteria for surgical treatment were pelvic pain, which prevented the patient from sitting down and standing on the second or third day after the injury, and severe pain during lateral loading of the pelvis. Therefore, the decision of treatment was based on clinical evaluation and examination under anesthesia (EUA) was not performed [17]. Non-operatively treated patients were mobilized on two crutches without weight-bearing on the affected side for six weeks. For operatively treated patients, full weight-bearing was allowed from the day after surgical stabilization.

Functional outcomes were evaluated using the Majeed pelvic score, which is the most widely used scale for measuring outcomes after pelvic fractures. In accordance with Majeed, functional results were graded as follows: >85 excellent; 70–84 good; 55–69 fair; and <55 poor [18]. Changes in quality of life were assessed using the 36-item Short Form Health Survey (SF-36) validated questionnaire. The SF-36 consists of eight domains: physical functioning (PF); role-physical (RP); bodily pain (BP); general health (GH); vitality (VT); social functioning (SF); role-emotional (RE); and mental health (MH). Each domain was scored on a scale from 0 to 100, with 100 representing the best possible score. In addition, the physical component summary (PCS) and mental component summary (MCS) were calculated [19]. Patients completed these questionnaires twice: first during hospitalization (regarding their pre-traumatic condition–timepoint I); and again 10 weeks after their injury during outpatient control (regarding their current condition–timepoint II).

Statistical analysis was performed using the R commander version 3.5.1. Figures are presented as mean ± standard deviation, and groups were compared using the Chi-squared test. For mean comparison, the Mann-Whitney U and Wilcoxon tests were used for non-parametric data, while the Student’s *t*-test and the paired sample *t*-test were used for parametric data. Differences were considered significant at *p* < 0.05.

## 3. Results

A total of 55 patients with B2.1 pelvic fractures, according to the AO/Tile classification, met the inclusion criteria, and were thus included in the final analysis. Of these 55, 42 (70.6%) patients were female and 13 (23.6%) were male, with an average age of 37.24 ± 13.78 years. The median (IQR) of ISS was 15.00 (10.00–18.00), and there were 21 (38.18%) patients with high injury severity (ISS ≥ 18). Concomitant injuries, predominantly fractures of other bones, were diagnosed in 31 (56.5%) patients. As a result of concomitant injuries, surgeries other than pelvic fixation were performed on 14 (25.5%) patients. At timepoint I (before their injury), the mean Majeed score was 97.98 ± 9.35, while the mean PCS and MCS scores of the SF-36 questionnaire were 56.26 ± 6.36 and 51.54 ± 6.68, respectively. The mean Majeed, PCS, and MCS scores at timepoint II (10 weeks after injury) were 64.76 ± 18.57, 36.64 ± 8.86, and 44.75 ± 10.93, respectively.

A total of 37 (67.3%) patients were treated operatively, and 18 (32.7%) were treated non-operatively. In the operative group, 23 patients were treated with anterior and posterior pelvic ring fixation, 13 patients were treated with posterior fixation only, and one patient was treated with external fixation of the anterior pelvic ring due to infection. Moreover, 7 patients developed surgical complications: 1 patient developed a wound infection; two patients developed screw migration; and 4 patients developed S1 neuropathy. No treatment-related complications were observed for non-operatively treated patients. A more detailed comparison between the groups of operatively and non-operatively treated patients can be found in Table 1. The only statistically significant differences between groups involved concomitant injuries and ISS, while the other characteristics remained similar.

Analysis of Majeed, PCS, and MCS scores in operative and non-operative treatment groups revealed that all scores in both groups were statistically significantly lower at timepoint II compared with timepoint I, with the exception of MCS score in the non-operative group, which was lower but did not reach the level of statistical significance (Table 2).

**Figure 2:**
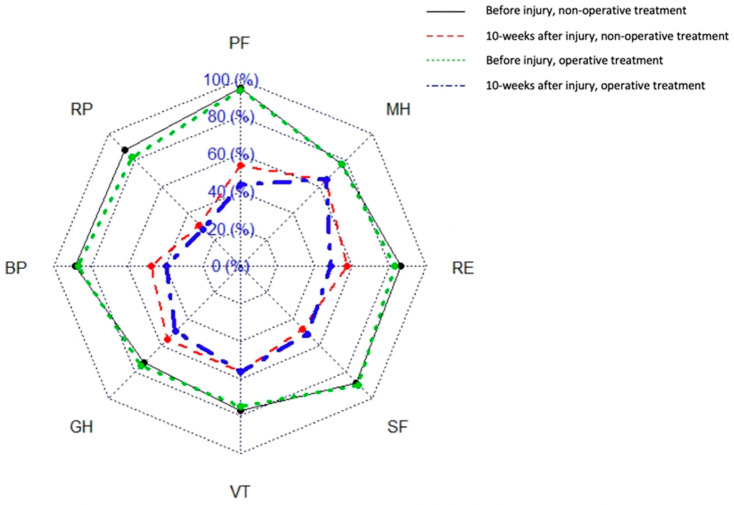
Percentage differences between results of SF-36 domains for operatively and non-operatively treated patients between timepoint I and timepoint II (PF—physical functioning; RP—role-physical; BP—bodily pain; GH—general health; VT—vitality; SF—social functioning; RE—role-emotional; and MH—mental health).

A detailed analysis of SF-36 domains and Majeed results was performed, and the change in each score between timepoints (Δ = timepoint II−timepoint I) was calculated. The analysis revealed that there were no statistically significant differences between the operative and non-operative groups of treatment (Table 3; Figure 2). However, tree analysis showed that the change in quality of life was greater for patients whose PCS had been above 57.16 and MCS above 45.22 before their injury. Moreover, because a significant proportion of all patients (38.18%) suffered a high severity injury, the results of SF-36 domains and Majeed score were also compared between patients with high and low injury severity. The only statistically significant difference between the results at timepoint I and timepoint II was found to concern social functioning in low injury severity patients: there was less of a reduction in social functioning for operatively than for non-operatively treated patients (−25.00 ± 29.80 vs. −44.64 ± 26.73, respectively) (Table 3).

## 4. Discussion

Our research showed that quality of life and pelvic function significantly decreased 10 weeks after suffering a pelvic fracture compared to pre-trauma state for both operated and non-operated patients. However, there were no statistically significant differences between the two treatment groups. It was found that, for patients with low injury severity, there was less of a reduction in social functioning for operatively than for non-operatively treated patients.

Most studies focus on evaluating long-term outcomes after pelvic injuries. We found only a few articles that evaluated the short-term outcomes of pelvic ring fractures. However, of these studies, only one article could be found which analyzed the short-term functional outcomes of the most common pelvic fractures—type B2. Lykomitros et al. found that, when compared to those who were treated operatively, patients with sacral fractures who were treated non-operatively achieved better scores in all of the domains of the SF-36 questionnaire. The authors explained this phenomenon by noting that non-operatively treated patients had fewer concomitant injuries, and the ISS had therefore been lower at the time of their initial evaluation [11]. Our study shows no differences among the domains of the SF-36 questionnaire regarding treatment method, except for the greater reduction of social functioning in patients with low injury severity who were treated non-operatively. We would like to point out that, in our research, operatively treated patients had more concomitant injuries and therefore a higher ISS at their initial evaluation.

Kokubo et al. evaluated the factors that correlated with unsatisfactory short-term (one-year follow-up) outcomes in patients who sustained unstable pelvic ring fractures. Non-operative therapy was one of the factors which showed a significant relationship with unsatisfactory short-term functional outcomes [20]. Unlike us, they did not distinguish type B fractures from type C fractures, and our study therefore produces the opposite results. However, analysis of the tables provided by Kokubo et al. reveals that there were no differences in functional outcomes between operated and non-operated patients after type B pelvic fractures in their study.

Höch et al. performed a retrospective analysis of operatively treated and non-operatively treated young patients after lateral compression type B2.1 pelvic ring fractures. They used the visual analogue scale (VAS) for pain alongside SF-36 and European Quality of Life 5-Dimensions (EQ-5D) questionnaires for the evaluation of outcomes, and the follow-up of patients lasted for at least one year postoperatively. They found that there were no significant differences regarding pain or quality of life between operatively treated and non-operatively treated patients. However, there was a significantly higher complication rate in the operatively treated group [13]. Their conclusion—that type B2.1 pelvic fractures should be treated non-operatively—is consistent with the results of our study.

Hagen et al. performed a retrospective analysis of 158 patients with LC-1 fractures treated in non-surgical and surgical settings. They found no evidence that the surgical stabilization of LC-1 pelvic fractures would reduce patients’ pain, decrease their use of narcotic analgesics, or reduce their time to mobilization [12]. However, the research of Tosounidis et al.—based on data of the German Pelvic Multicenter Studies I and III on the epidemiology and treatment of pelvic ring injuries—provided very controversial conclusions, stating that the surgical stabilization of LC-1 pelvic fractures reduced the length of hospital stay and significantly reduced pain and analgesic requirements during the immediate post-injury period [14].

Papakostidis et al. performed a systematic review of the English literature over the last 30 years with the purpose of finding a correlation between the clinical outcomes of different types of pelvic ring injuries and the methods of their treatment. They found that fixation of all the injured elements of the pelvic ring yielded better radiological results and lower malunion rates compared with non-operatively treated pelvic injuries. However, they did not find clear advantages to either method when comparing functional outcomes between operatively treated and non-operatively treated patients [21].

Our study has several limitations that must be taken into account. Firstly, this is a single-center study that only involves patients with pelvic fractures from the largest region of our country. Secondly, the study was not randomized regarding the treatment method for comparison and evaluation of outcomes after type B2.1 pelvic fracture, and the results of this study should therefore be interpreted with caution. Nevertheless, our findings may provide a reference for future randomized controlled trials.

## 5. Conclusions

We observed that operative treatment of B2.1 type pelvic fractures for young patients is not superior to non-operative in the short term, because the functional outcomes and quality of life are similar in both groups. We found that, for patients with low injury severity, there was less of a reduction in social functioning when they had been treated operatively.

## Figures and Tables

**Table 1:** Comparison between the operatively and non-operatively treated groups. Majeed, PCS (physical component summary), MCS (mental component summary), and age are presented as mean ± SD (standard deviation). ISS (Injury Severity Score) is presented as median (IQR). Majeed I, PCS I, and MCS I relate to the results at timepoint I, before the injury; whereas Majeed II, PCS II, and MCS II relate to timepoint II, 10 weeks after the injury. Figures in bold represent statistically significant *p* values.

	Treatment Group		*p* Value
	Operative (*N* = 37)	Non-Operative (*N* = 18)	
Age	35.84 ± 12.22	40.11 ± 16.56	0.404
Female	27 (73.0%)	15 (83.3%)	0.510
Concomitant injuries	25 (67.6%)	6 (33.3%)	**0.016**
Other surgeries	12 (32.4%)	2 (11.1%)	0.110
High injury severity (ISS ≥ 18)	17 (45.9%)	4 (22.2%)	0.089
ISS	17.00 (11.00–18.00)	10.50 (9.00–17.25)	**0.011**
Majeed I	97.50 ± 11.14	99.78 ± 0.94	0.629
PCS I	56.04 ± 7.89	55.73 ± 4.51	0.477
MCS I	51.89 ± 7.30	51.05 ± 7.09	0.799
Majeed II	64.61 ± 19.63	67.44 ± 15.17	0.516
PCS II	35.88 ± 9.21	38.99 ± 7.18	0.087
MCS II	44.72 ± 10.92	45.00 ± 10.70	0.875

**Table 2:** Majeed, PCS (physical component summary), and MCS (mental component summary) results (presented as mean ± SD (standard deviation)) at timepoint I and timepoint II for operatively and non-operatively treated patients. Figures in bold represent statistically significant *p* values.

Treatment Group		Timepoint I	Timepoint II	*p* Value
Operative (*N* = 37)	Majeed	97.50 ± 11.14	64.61 ± 19.63	**<0.001**
	PCS	56.04 ± 7.89	35.88 ± 9.21	**<0.001**
	MCS	51.89 ± 7.30	44.72 ± 10.92	**0.001**
Non-operative (*N* = 18)	Majeed	99.78 ± 0.94	67.44 ± 15.17	**<0.001**
	PCS	55.73 ± 4.51	38.99 ± 7.18	**<0.001**
	MCS	51.05 ± 7.09	45.00 ± 10.70	0.071

**Table 3:** Differences in SF-36 domains and Majeed results for operatively and non-operatively treated patients between timepoints (Δ = timepoint II−timepoint I). In the lower part of the table, SF-36 and Majeed results are presented depending on the injury severity. (PF—physical functioning; RP—role-physical; BP—bodily pain; GH—general health; VT—vitality; SF—social functioning; RE—role-emotional; MH—mental-health; PCS—physical component summary; and MCS—mental component summary). Figures in bold represent statistically significant *p* values.

	SF-36 Domains and Majeed	Treatment Group		*p* Value
		Operative (*N* = 37)	Non-Operative (*N* = 18)	
All patients (*N* = 55)	ΔPF	−50.54 ± 29.15	−41.67 ± 21.21	0.258
	ΔRP	−54.39 ± 31.42	−56.60 ± 21.70	0.725
	ΔBP	−47.30 ± 29.02	−40.72 ± 25.58	0.445
	ΔGH	−26.38 ± 25.82	−17.06 ± 15.60	0.151
	ΔVT	−18.24 ± 20.75	−20.83 ± 23.29	0.899
	ΔSF	−38.51 ± 35.52	−40.97 ± 28.05	0.684
	ΔRE	−34.46 ± 30.31	−28.70 ± 27.14	0.499
	ΔMH	−11.22 ± 21.81	−12.50 ± 20.95	0.850
	ΔPCS	−19.45 ± 9.95	−19.36 ± 7.88	0.687
	ΔMCS	−6.38 ± 11.04	−7.23 ± 10.86	0.816
	ΔMajeed	−34.08 ± 18.95	−31.44 ± 14.41	0.542
High injury severity (ISS ≥ 18) (*N* = 21)	ΔPF	−57.65 ± 32.94	−37.50 ± 21.02	0.243
	ΔRP	−61.76 ± 30.37	−48.44 ± 7.86	0.114
	ΔBP	−55.06 ± 29.79	−36.25 ± 16.58	0.301
	ΔGH	−26.06 ± 24.95	−23.50 ± 14.48	0.929
	ΔVT	−22.06 ± 16.85	−12.50 ± 16.14	0.340
	ΔSF	−54.41 ± 35.89	−28.13 ± 32.87	0.221
	ΔRE	−32.84 ± 30.54	−25.00 ± 24.53	0.651
	ΔMH	−13.82 ± 19.73	−6.25 ± 12.50	0.558
	ΔPCS	−18.84 ± 10.15	−15.31 ± 5.18	0.244
	ΔMCS	−7.64 ± 10.18	−3.51 ± 6.96	0.474
	ΔMajeed	−35.29 ± 18.82	−21.75 ± 10.47	0.139
Low injury severity (ISS < 18) (*N* = 34)	ΔPF	−44.50 ± 24.76	−42.86 ± 21.90	0.806
	ΔRP	−48.13 ± 31.68	−58.93 ± 23.98	0.439
	ΔBP	−40.70 ± 27.37	−42.00 ± 28.01	0.958
	ΔGH	−26.65 ± 27.18	−15.21 ± 15.92	0.161
	ΔVT	−15.00 ± 23.51	−23.21 ± 24.93	0.470
	ΔSF	−25.00 ± 29.80	−44.64 ± 26.73	**0.048**
	ΔRE	−35.83 ± 30.84	−29.76 ± 28.63	0.623
	ΔMH	−9.00 ± 23.71	−14.29 ± 22.86	0.427
	ΔPCS	−19.96 ± 10.01	−20.51 ± 8.28	0.986
	ΔMCS	−5.31 ± 11.87	−8.29 ± 11.73	0.363
	ΔMajeed	−33.05 ± 19.48	−34.21 ± 14.47	0.889

## Data Availability

The data presented in this study are available on request from the corresponding author. The data are not publicly available due to ethical restrictions.
